# Hydroxylation decoration patterns of flavonoids in horticultural crops: chemistry, bioactivity, and biosynthesis

**DOI:** 10.1093/hr/uhab068

**Published:** 2022-01-20

**Authors:** Yilong Liu, Jiafei Qian, Jiajia Li, Mengyun Xing, Donald Grierson, Chongde Sun, Changjie Xu, Xian Li, Kunsong Chen

**Affiliations:** 1 Zhejiang Provincial Key Laboratory of Horticultural Plant Integrative Biology, Zhejiang University, Hangzhou 310058, China; 2 Shandong (Linyi) Institute of Modern Agriculture, Zhejiang University, Linyi 276000, China; 3 Plant and Crop Sciences Division, School of Biosciences, Sutton Bonington Campus, University of Nottingham, Loughborough LE12 5RD, UK

## Abstract

Flavonoids are the most widespread polyphenolic compounds and are important dietary constituents present in horticultural crops such as fruits, vegetables, and tea. Natural flavonoids are responsible for important quality traits, such as food colors and beneficial dietary antioxidants, and numerous investigations have shown that intake of flavonoids can reduce the incidence of various non-communicable diseases. Analysis of the thousands of flavonoids reported so far has shown that different hydroxylation modifications affect their chemical properties and nutritional values. These diverse flavonoids can be classified based on different hydroxylation patterns in the B, C, and A rings and multiple structure–activity analyses have shown that hydroxylation decoration at specific positions markedly enhances their bioactivities. This review focuses on current knowledge concerning hydroxylation of flavonoids catalyzed by several different types of hydroxylase enzymes. Flavonoid 3′-hydroxylase (F3′H) and flavonoid 3′5′-hydroxylase (F3′5′H) are important enzymes for the hydroxylation of the B ring of flavonoids. Flavanone 3-hydroxylase (F3H) is key for the hydroxylation of the C ring, while flavone 6-hydroxylase (F6H) and flavone 8-hydroxylase (F8H) are key enzymes for hydroxylation of the A ring. These key hydroxylases in the flavonoid biosynthesis pathway are promising targets for the future bioengineering of plants and mass production of flavonoids with designated hydroxylation patterns of high nutritional importance. In addition, hydroxylation in key places on the ring may help render flavonoids ready for degradation, and the catabolic turnover of flavonoids may open the door for new lines of inquiry.

## Introduction

**Figure 1:**
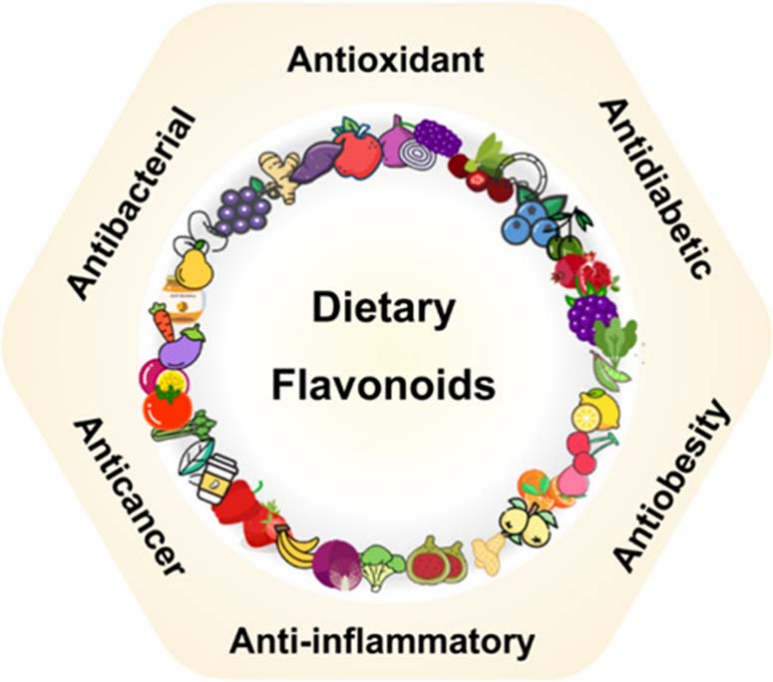
Health-promoting bioactivities of flavonoids in horticultural crops.

Flavonoids are the most widespread polyphenolic compounds, characterized by possession of at least one aromatic ring with one or more hydroxyl groups attached. Current dietary guidance from the World Health Organization recommends people should consume at least 400 g, i.e. five portions, of fruits and vegetables per day for their optimum health. Besides vitamins, minerals, and dietary fiber, such horticultural crops have the advantage of accumulating high amounts of polyphenols, especially flavonoids [1]. These substances *in planta* are generated as the products of plant antioxidative defense systems activated in rapid response to both biotic and abiotic stresses and are responsible for important quality traits, such as color, in horticultural crops [2]. Moreover, over recent years, numerous epidemiological and clinical studies have shown that such flavonoids also exhibit strong antioxidant properties *in vitro* and *in vivo*, as well as antidiabetic, antiobesity, anti-inflammatory, anticancer, and antibacterial activities [1, 3, 4] (Fig. 1). Hence, flavonoid-abundant foods might be nature’s bountiful gifts to humankind for their excellent health-promoting benefits.

**Figure 2:**
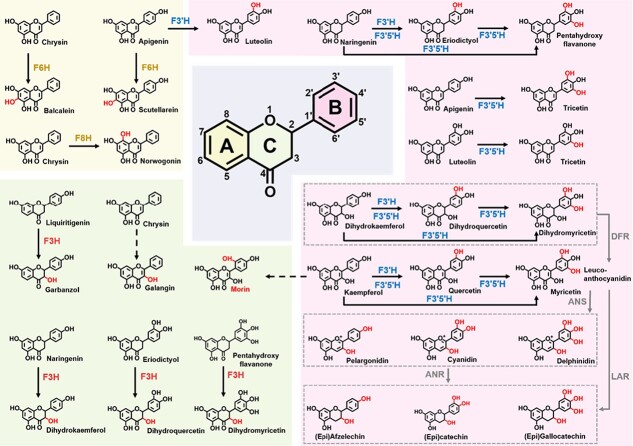
Key hydroxylases that participate in hydroxylation decoration during flavonoid biosynthesis. F3H, flavanone 3-hydroxylase; F3′H, flavonoid 3′-hydroxylase; F3′5′H, flavonoid 3′,5′-hydroxylase; F6H, flavone 6-hydroxylase; F8H, flavone 8-hydroxylase; DFR, dihydroflavonol 4-reductase; ANS, anthocyanidin synthase; ANR, anthocyanidin reductase; LAR, leucoanthocyanidin reductase.

There are >8000 different flavonoids identified from plants [5]. Different modifications, such as hydroxylation, glycosylation, methylation, and acylation, play important roles in generating such diversity of flavonoids, and hydroxylation is the most frequently occurring modification in natural flavonoids (Fig. 2). Hydroxylation of flavonoids improves the chemical solubility and stability of an array of flavonoids and is also correlated with their bioactive properties. For instance, a hydroxylated B ring is crucial for the antioxidant properties of flavonoids [6]. An increased degree of hydroxylation of flavonoids is associated with stronger inhibitory effects on α-glucosidase or α-amylase activities [7, 8]. Inhibition of aldose reductase activity was also found to be remarkably enhanced by the hydroxylation of specific positions, such as the C3′ and C4′ of the B ring of flavonoids [9]. Taken together, the data show that different hydroxylation decoration patterns of flavonoids may affect their chemical properties as well as their bioactivities. So far, there have been several review papers describing the structures and biosynthesis of flavonoids and the introduction of various decorations, such as glycosylation [10–12], methylation [13, 14], and acylation [15]. However, to date, there is no systematic review on the hydroxylation modification of flavonoids, particularly in horticultural crops.

This review focuses on current knowledge concerning hydroxylation decoration of flavonoids and its effects on their chemical and nutritional value. Diverse dietary flavonoids are further classified based on different hydroxylation patterns in the B, C, and A rings of flavonoids and a comparison is made of bioactivities of flavonoids that differ only by hydroxylation patterns. Particular attention is paid to key hydroxylases in the flavonoid biosynthesis pathway, with the objective of future bioengineering-targeted bioactivities in plant resources and mass production of flavonoids with desirable hydroxylation patterns. We also discuss the degradation of natural flavonoids and the relationship between hydroxylation modification and flavonoid catabolism *in planta*, which requires further investigation in the future.

## Divergent hydroxylation patterns and distribution of flavonoids

Flavonoids consist of two aromatic rings (A and B rings) connected by a three-carbon bridge (C ring) containing an embedded oxygen atom, abbreviated as C6-C3-C6 (Fig. 2). According to the oxidative status and hydroxylation degree of the C ring and the connection position of the B ring, flavonoids can be grouped into different subclasses, including flavonols, anthocyanidins, flavones, flavanones, flavanols, and isoflavones (Table 1). Each subclass of flavonoids can be analyzed based on its hydroxylation patterns.

**Table 1:** Divergent hydroxylation patterns of flavonoids and their distribution in horticultural crops.

${\includegraphics{\bwartpath uhab068t1}} $

Different flavonoids are listed within each subclass according to the position of -OH in the order of A ring, followed by B ring with increasing number of hydroxyl groups. Compounds are listed based on the relative amount of each compound, from high to relatively low, in different horticultural crops.

### Hydroxylation and distribution of flavonols

Flavonols are the most widespread subclass of flavonoids and possess the 3-OH and 4-oxo groups on the C6-C3-C6 backbone. Their diversity originates from different substitution of the phenolic -OH groups in both the A and the B ring (Table 1).

The flavonol fisetin is hydroxylated at C7, C3, C3′, and C4′ and is found mainly in strawberry, apple, persimmon, onion, and grape [16]. Galangin, kaempferol, morin, quercetin, and myricetin are common flavonols having the same structures on the A and C rings with hydroxyl groups attached at C5, C7, and C3, but with different hydroxylation modes on their B ring (Table 1). Specifically, galangin, which is a major active component in the root of galangal [17] and is also found in propolis produced from plants by bees [18], has no -OH on the B ring. Kaempferol, with an -OH group substituted at C4′, is abundant in green leafy vegetables such as spinach and kale, and berries, including mulberry and strawberry [19, 20]. Morin also has two -OH groups on the B ring at the C2′ and C4′ positions and is found mainly in mulberry, guava, grape, and fig [21]. Quercetin, which is hydroxylated at both C3′ and C4′, is present in a high concentration in onion, chili pepper, apple, Chinese bayberry, mulberry, and apricot [19, 20, 22]. Notably, myricetin has three hydroxyl groups at C3′, C4′, and C5′. It was originally isolated from the bark of Chinese bayberry [23] and is also present at a relatively high level in its fruit [22]. Myricetin is also widely distributed in many other berries, such as strawberry, blackberry, and blueberry, as well as vegetables like spinach and cauliflower [20, 24]. Quercetagetin which has three -OH groups on the A ring (C5, C6, and C7) is found mainly in *Tagetes* [25].

### Hydroxylation and distribution of anthocyanidins

Anthocyanins are the glycoside conjugates of anthocyanidins (Table 1) and constitute a group of natural pigments that confer a wide spectrum of colors, varying from orange, salmon pink, red, magenta, and violet to dark blue in many fruits, colored leafy vegetables, and tubers. The majority of anthocyanins reported are based on three common anthocyanidins, i.e. pelargonidin, cyanidin, and delphinidin [26]. These three compounds share the same chemical structures at the A and C rings with a characteristic flavylium cation and three -OH groups attached at C5, C7, and C3. They have different degrees of hydroxylation on the B ring (Table 1) and, interestingly, their colors seem to gradually deepen with increasing hydroxylations, i.e. orange/red (pelargonidin), red/magenta (cyanidin), and violet/blue (delphinidin), respectively [27].

Pelargonidin, hydroxylated at C4′, is abundant in strawberry [28], and distributed in red radish, potato, and banana [29]. Cyanidin, with two hydroxyl groups at C3′ and C4′ of the B ring, is the most common anthocyanidin. It is especially rich in berries such as Chinese bayberry [22], elderberry, chokeberry, blackberry, black mulberry, and cherry [28, 29]. Cyanidin is also ubiquitously found in other fruits, like apple, peach, pear, and fig, as well as colored vegetables like red onion and red cabbage [29]. Delphinidin has three hydroxyl groups at C3′, C4′, and C5′ of the B ring, the major sources of which were dark-colored foods such as bilberry, highbush blueberry, blackcurrant, eggplant, etc. [28, 29]. It is also found in passion fruit, green bean, and pomegranate [29].

### Hydroxylation and distribution of flavones

Flavones lack the hydroxyl group at C3 compared with the skeleton of flavonols, but have a wide range of hydroxylation patterns on the A and B rings. Typically, chrysin, apigenin, luteolin, and tricetin are common flavones that all have hydroxylation at C5 and C7 but differ from each other by the substitution of -OH groups on their B rings (Table 1). Chrysin has no hydroxyl on the B ring and is found in honey, propolis, and passion fruit [30]. Apigenin (4′-OH) is particularly abundant in parsley and dried flowers of chamomile, and exists in some other vegetables, such as celery, broccoli, spices like thyme, and fruits such as cherry, olive, and legumes [31]. Major food sources of luteolin (3′,4′-OH) include bird chili [32], celery, broccoli, parsley, chrysanthemum flowers, onion leaves, sweet bell pepper, and carrot [33]. Tricetin (3′,4′,5′-OH) is found mainly in Myrtaceae pollen and *Eucalyptus* honey [34]. In addition, other flavones, such as baicalein or norwogonin, show additional hydroxylations on carbons 6 or 8, based on the structure of chrysin; and scutellarein has an additional -OH group substituted at the C4′ of baicalein or C6 of apigenin (Table 1). These three flavones specifically exist in *Scutellaria baicalensis* and *Scutellaria barbata* [35].

### Hydroxylation and distribution of flavanones

Flavanones that have a chiral carbon at C2 are the hydrogenated derivatives of flavones. The flavanone liquiritigenin has a C7-hydroxyl on the A ring and C4′-hydroxyl on its B ring and is particularly prevalent in licorice [36] (Table 1). Flavanones including pinocembrin, naringenin, eriodictyol, and 5,7,3′,4′,5′-pentahydroxy flavanone all have dihydroxyl groups at C5 and C7, with the structural difference involving sequential increases in B-ring hydroxylation (Table 1). Pinocembrin has no -OH substitution on the B ring and in the (2*S*)-form is widely distributed in propolis and *Glycyrrhiza glabra* [37]. Naringenin (4′-OH) occurs extensively in *Citrus* fruits like grapefruit, orange, and lemon [38]. Additionally, eriodictyol (3′,4′-OH) is also widely distributed in *Citrus* fruits such as bitter orange, mandarin, tangerine, and lemon, as well as peanuts and loquats [39]. Pentahydroxy flavanones also exist in some horticultural crops. For instance, 5,7,3′,4′,5′-pentahydroxy flavanone has been detected in *Helichrysum bracteatum* [40].

### Hydroxylation and distribution of flavanols

Compared with other subclasses of flavonoids, flavanols are characterized by the absence of a double bond between C2 and C3 and have no C4 carbonyl in the C ring (Table 1). Therefore, two chiral carbons (C2 and C3) exist in flavanols and the fixed hydroxylation at C3 means each of them has four possible diastereoisomers. For instance, catechin, the most typical monomeric flavanol, exists in four forms, i.e. (−)-catechin (2*S*,3*R*), (+)-catechin (2*R*,3*S*), (−)-epicatechin (2*S*,3*S*), and (+)-epicatechin (2*R*,3*R*). Of these, (+)-catechin and (−)-epicatechin are most commonly found in horticultural crops, especially in tea leaves [41, 42]. Catechin has four hydroxyl groups in the basic skeleton of flavan-3-ol at C5, C7, C3′, and C4′, respectively. Afzelechin, which lacks the 3′-OH of catechin, is present in sour jujube [43], cowpea [44], and peanut seed skin [45]. Gallocatechin has one more -OH substitution at C5′ of catechin, and is present at significant levels in tea [41, 42]. The 3-OH on the C ring of catechins is usually esterified with gallic acid, thereby forming the gallated catechins such as epigallocatechin gallate, epicatechin gallate, and catechin gallate.

### Hydroxylation and distribution of isoflavones

Unlike other flavonoids, the isoflavone B ring is attached at C3 rather than C2 (Table 1). Isoflavones such as 4',7-dihydroxyisoflavone (daidzein) and 4′,5,7-trihydroxyisoflavone (genistein), are specifically accumulated in legumes, especially soybeans [46]. Thus, soybean-based foods such as tofu, soy milk, and soy yoghurt are excellent dietary sources of isoflavones.

## Bioactivities of flavonoids influenced by divergent hydroxylation patterns

### For the B ring

With the increasing attention paid to the healthcare benefits of natural products, there are more and more studies focusing on the structure–activity relationships between the different chemical modifications of flavonoids and their bioactivities [47, 48]. Plenty of studies have shown that hydroxylation decoration at specific positions of flavonoids markedly enhances efficacy of their bioactivity (Table 2).

**Table 2:** Different bioactivities of flavonoids based on their divergent hydroxylation patterns.

	**Compounds**	**Bioactivities**	**References**
	**Kaempferol → quercetin → myricetin**	Scavenging hydroxyl radicals ↑	49
	4′-OH → 3′,4′-OH → 3′,4′,5′-OH	Trapping ability of ACR ↑	50
		Protection activity against light-mediated photoreceptor damage ↑	51
		Inhibition of α-glucosidase ↑	52, 53
		Inhibits EGF-induced cell transformation ↑	54
		Constituent of active packaging films containing flavonols ↑	55
		Induction of apoptosis in tumor cells ↑	56
		Inhibition of tyrosinase ↑	57
**B ring**	**Kaempferol → morin**	Scavenging hydroxyl radicals ↑	49
	4′-OH → 2′,4′-OH	Inhibition of formation of fAβ ↑	58
		ACR trapping efficiency ↑	50
	**Galangin → kaempferol** No hydroxyl → 4′-OH	ACR trapping efficiency ↑	50
	**Pelargonidin → cyanidin → delphinidin**	Superoxide radical- and peroxynitrite-scavenging activity ↑	59, 60
	4′-OH → 3′,4′-OH → 3′,4′,5′-OH	Inhibition of lipid peroxidation ↑	61
		Antioxidant activities ↑	60
	**C3G → D3G**	Stimulation of insulin secretion ↑	64
	3′,4′-OH → 3′,4′,5′-OH	Inhibition of viability of cancer cells ↑	65
	**Compounds**	**Bioactivities**	**References**
	**Chrysin → apigenin → luteolin** No hydroxyl → 4′-OH → 3′,4′-OH	Induction of apoptosis in tumor cells ↑	66
		Inhibition of proteasome activity ↑	66, 67
		MMP inhibitory effect ↑	68
**B ring**		Reactive oxygen scavenging activities ↑	68
		Inhibition of pro-inflammatory cytokine production ↑	69
	**Pinocembrin → naringenin → eriodictyol**	Antioxidant activity ↑	70, 71
	No hydroxyl → 4′-OH → 3′,4′-OH	Inhibition of the phosphorylation of PKCδ ↑	70
		Inhibition of the formation of AGEs ↑	72
	**Tricetin → myricetin** 3H → OH	Pro- and antioxidant activity ↑	73
**C ring**	**Apigenin→ kaempferol** 3H → OH	Inhibition of α-glucosidase or α-amylase ↑	74
		Anti-adipogenic action ↑	75
	**Fisetin → quercetin** 5H → OH	Membrane binding ↑	76
**A ring**	**Daidzein → genistein** 5H → OH	Antioxidant activity ↑	77
		Improves glucose tolerance↑	78

fAβ, β-amyloid fibrils; C3G, cyanidin 3-*O*-glucoside; D3G, delphinidin 3-*O*-glucoside; ACR, acrolein; EGF, epidermal growth factor; MMP, matrix metalloproteinease; PKCδ, protein kinase Cδ; AGEs, advanced glycation end products.

The flavonols kaempferol (4′-OH), quercetin (3′,4′-OH), and myricetin (3′,4′,5′-OH) are a typical group of flavonoids with the same structures for the A and C rings but have different hydroxylation modes on the B ring. Results have shown that quercetin has stronger ^•^OH scavenging properties than kaempferol, and myricetin is the most powerful hydroxyl radical scavenger, indicating that radical scavenging activity increases with the growing number of -OH groups on the aromatic B ring [49] (Table 2). Furthermore, their acrolein-trapping efficiency decreases in the order of myricetin > quercetin > kaempferol [50]. Exposure to blue light for 20 hours induced the death of ~75% of the photoreceptors in bovine retinal cell cultures, while myricetin conferred ~100% protection against light-mediated damage to photoreceptors, whereas quercetin showed relatively poor protective activity, and kaempferol was inactive [51]. Among these three flavonols, myricetin showed the strongest inhibitory effects on either α-glucosidase [52, 53] or epidermal growth factor-induced cell transformation in JB6 P^+^ cells [54], followed by quercetin and kaempferol. Such flavonols can also be individually mixed with a chitosan-based matrix to develop active packaging films, and myricetin showed the strongest intermolecular interactions with the film matrix, due to the greater number of hydroxyl groups on the B ring [55]. Hence, films containing myricetin have the most satisfactory mechanical properties and the highest ability to provide a barrier to water vapor and oxygen [55]. In addition, studies have reported that myricetin is more effective than quercetin at inhibiting the induction of apoptosis in prostate cancer cell line PC-3 [56]. Also, quercetin has a stronger tyrosinase inhibitory effect than kaempferol [57].

Morin (2′,4′-OH), with an additional hydroxyl at the C2′ position of kaempferol, shows enhanced hydroxyl radical scavenging activity [49]. It has a greater ability to inhibit β-amyloid fibril formation from amyloid β-peptide and destabilizes preformed β-amyloid fibrils, demonstrating its greater potential for the prevention and control of Alzheimer’s disease [58]. Galangin, another flavonol with no hydroxylation on the B ring, as well as morin and kaempferol, all show significant scavenging ability for acrolein, and their trapping efficiency increases in the order galangin < morin < kaempferol [50].

The activities of three anthocyanidins in O_2_^•−^ and ONOO^−^ scavenging and inhibition of lipid peroxidation have been ranked in the order pelargonidin (4′-OH) < cyanidin (3′,4′-OH) < delphinidin (3′,4′,5′-OH), indicating that the increasing number of -OH groups present on the B ring enhances the antioxidant activities of anthocyanidins [59–61] (Table 2). As mentioned previously, the degree of hydroxylation is related to the gradually deepening colors of these three anthocyanidins and, therefore, dark-colored fruits and vegetables may show greater health benefits [62, 63]. Similarly, compared with cyanidin 3-*O*-glucoside, delphinidin 3-*O*-glucoside showed enhanced ability to stimulate insulin secretion [64], and greater ability to inhibit the viability of cancer cells such as HCT 116 cells [65] (Table 2).

Chrysin (no hydroxyl at the B ring), apigenin (4′-OH), and luteolin (3′,4′-OH) belong to group of flavones with increasing numbers of hydroxyl substituents on the B ring. Their apoptosis-inducing potencies in tumor cells [66], proteasome inhibitory activities [66, 67], matrix metalloproteinase (MMP) inhibition effects, and scavenging reactive oxygen capacities [68] are in the order of luteolin > apigenin > chrysin (Table 2). Also, compared with chrysin, apigenin showed a stronger ability to inhibit the production of pro-inflammatory cytokines stimulated by lipopolysaccharide in human peripheral blood mononuclear cells [69].

There are three kinds of flavanones sharing similar chemical structures that differ only in the number of hydroxyl groups on their B ring, namely, pinocembrin (no hydroxyl), naringenin (4′-OH), and eriodictyol (3′,4′-OH). Studies have demonstrated that eriodictyol showed markedly higher antioxidant capacity than naringenin and pinocembrin, as reflected in its stronger inhibition of the phosphorylation of protein kinase Cδ (PKCδ) and p47 in murine macrophage RAW264.7 cells [70, 71] (Table 2). Docking analysis showed that eriodictyol has the most favorable binding with the phorbol ester binding site of PKCδ, which may be due to its greater number of hydroxyl groups on the B ring conferring the ability to form hydrogen bonds with the kinase, and thus strengthened the interaction [70]. In addition, eriodictyol showed stronger inhibitory effects on the formation of advanced glycation end products than naringenin, which may be due partly to its additional -OH in the 3′ position of the B ring [72].

### Other ring modifications

Apart from the hydroxylations on the B ring, hydroxyl substituents present on either the C or A ring may also affect the biological activity of flavonoids (Table 2). For instance, myricetin shows enhanced pro- and antioxidant effects compared with tricetin, which may be due to the positive mesomeric effect of the enolic 3-hydroxy group on the C-ring [73]. Kaempferol has a stronger inhibitory effect on α-glucoside and α-amylase with the substitution of an additional hydroxyl group at the C3 position of apigenin [74]. Moreover, treating preadipocytes with kaempferol reduces triacylglycerol content, whereas apigenin treatment has no such anti-adipogenic action [75]. Quercetin (5-OH) has a significantly higher membrane binding constant than fisetin (5-H), indicating that the hydroxylation at the C5 position of the A ring plays an important role in membrane-dependent processes associated with their biological activities [76]. The isoflavone genistein (5-OH) shows stronger antioxidant activity than daidzein (5-H) [77] and supplementing with genistein significantly improved glucose tolerance in diabetic *db*/*db* mice, which was not observed in the daidzein-supplemented group [78].

## Key hydroxylases in the biosynthesis pathways of flavonoids

Flavonoids are synthesized through the phenylpropanoid metabolic pathway, initiated by transformation of phenylalanine into 4-coumaroyl-CoA. This is combined with malonyl CoA, and naringenin is then synthesized by the decarboxylation and cyclization reactions catalyzed by chalcone synthase (CHS) and chalcone isomerase. Further, naringenin is converted by flavanone 3-hydroxylase (F3H) and other hydroxylases, including flavonoid 3′-hydroxylase (F3′H) and flavonoid 3′,5′-hydroxylase (F3′5′H), to produce different dihydroflavonols, which can form leucoanthocyanidins catalyzed by dihydroflavonol 4-reductase (Fig. 2). Subsequently, leucoanthocyanidins are converted into anthocyanidins or flavanols by the action of anthocyanidin synthase or leucoanthocyanidin reductase, respectively. Anthocyanidins can also be transformed to flavanols by the catalysis of anthocyanidin reductase.

The basic hydroxylation at C5, C7, and C4' of common flavonoids is shown for naringenin, while additional hydroxyl groups can also occur at C3, C3', C5', C6, and C8 positions (in Table 1). Hydroxylases play a vital role in the biosynthesis of hydroxylated flavonoids and F3′H and F3′5′H are important enzymes for the hydroxylation of the B ring of flavonoids (Fig. 2). F3H is key for the hydroxylation of the C ring and flavone 6-hydroxylase (F6H) and flavone 8-hydroxylase (F8H) are key enzymes for the hydroxylation of the A ring (Fig. 2).

### Flavonoid 3′-hydroxylase

F3′H (EC 1.14.13.21) is a cytochrome P450 (CYP450)-dependent monooxygenase requiring NADPH as a cofactor. It catalyzes the hydroxylation of flavanones, flavones, dihydroflavonols, and flavonols at the C3′ position of the B ring to their 3′,4′-hyroxylated states (Fig. 2). *F3*′*H* was first cloned from petunia (*Petunia hybrida*) [79], and then isolated from various resources such as soybean (*Glycine max*) [80], grapevine (*Vitis vinifera*) [81], sorghum (*Sorghum bicolor*) [82], apple (*Malus* × *domestica*) [83], strawberry (*Fragaria* × *ananassa*) [84], rice (*Oryza sativa*) [85], and tea (*Camellia sinensis*) [86] (Table 3).

**Table 3:** Transgenic analysis of the function of plant hydroxylase genes in the formation of flavonoids.

**Gene**	**Gene source**	**Target plant**	**Methods**	**Impact**	**References**
***F3*** ′***H***	Grapevine	Petunia	Overexpression (OE)	Quercetin ↑; peonidin ↑	81
		Grapevine	Gene silencing	Peonidin ↓; seed tannin ↓	89
	Sorghum	Arabidopsis *tt7* mutant	OE	Quercetin ↑; cyanidin ↑; condensed tannin ↑	82
	Apple	Arabidopsis *tt7* mutant	OE	Cyanidin ↑; pelargonidin ↑; quercetin ↑	83
		Tobacco	OE	Cyanidin ↑; quercetin ↑	
	*Ginkgo biloba* L.	*Populus*	OE	Epigallocatechin ↑; gallocatechin ↑; catechin ↑	90
***F3*** ′***5***′***H***	Potato	Potato	OE	Petunidin ↑	93
			Gene silencing	Anthocyanins↓; kaempferol ↑	98
	Pea	Pea	Gene silencing	Delphinidin↓; petunidin↓	95
	Grapevine	Petunia	OE	Malvidin ↑; quercetin ↑	81
	Tea	Tobacco	OE	Delphinin ↑; cyanidin ↑	96
***F3H***	Apple	Apple	Gene silencing	Flavanones ↑	109
	*Lycium chinense*	Tobacco	OE	Flavanols ↑	104
	Tea	Arabidopsis	OE	Flavonols ↑; oligomeric proanthocyanins ↑	105
		Tobacco	OE	Flavanols ↑	110
	Tomato	*are* Tomato mutant	OE	Anthocyanins ↑; flavonols ↑	111
	Soya bean	Soya bean	CRISPR/Cas9	Isoflavone ↑	113
***F6H***	*Scutellaria baicalensis*	*S. baicalensis*	Gene silencing	Baicalein↓; baicalin↓	118
		Arabidopsis	OE	Baicalein ↑	
***F8H***	*S. baicalensis*	*S. baicalensis*	Gene silencing	Wogonin↓; wogonoside↓	118
		Arabidopsis	OE	Norwogonin ↑	

**Figure 3:**
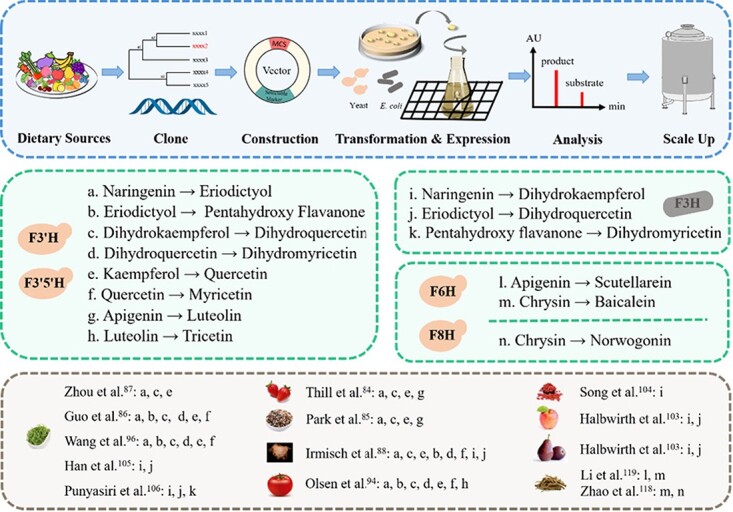
Biofactory scale-up production of desirable hydroxylated flavonoids. Pentahydroxy flavanone in this figure specifically refers to 5,7,3′,4′,5′-pentahydroxy flavanone.

Heterologous expression in yeast and analysis of F3′Hs from divergent plant species showed significant differences in substrate specificity and catalytic properties (Fig. 2). For instance, F3′H isolated from either tea [86, 87] or montbretia (*Crocosmia* × *crocosmiiflora*) [88] catalyzed the introduction of a hydroxyl group at the C3′ position of naringenin, dihydrokaempferol, and kaempferol to produce eriodictyol, dihydroquercetin, and quercetin, respectively (Fig. 3). Similarly, F3′H from strawberry (*Fragaria* sp.) showed high specificity for naringenin, dihydrokaempferol, and kaempferol, while apigenin was just a minor substrate [84]. However, F3′H encoded by *CYP75B4* from rice grain preferred apigenin to other substrates, leading to the formation of luteolin [85] (Fig. 3). Interestingly, *CYP75B3*, another rice *F3*′*H* gene, encodes an enzyme with a higher preference for kaempferol, and CYP75B3 from black rice showed around 2-fold increased catalytic efficiency with naringenin and dihydrokaempferol compared with the enzyme from either white or red rice [85]. It is also possible that different F3′H family members isolated from the same species might also have different catalytic functions.

Transgenic analysis has also been used to verify the functionality of F3′H from different plant sources (Table 3). Heterologous overexpression of grapevine *VvF3*′*H* in the petunia *ht1* mutant showed a significant accumulation of 3′,4′-hydroxylated flavonoids, including peonidin, an *O*-methylated anthocyanidin derived from cyanidin, and quercetin in the transgenic flowers [81]. In contrast, transgenic grapevines with the *F3*′*H* gene silenced had obviously decreased contents of peonidin as well as seed tannins [89]. Transgenic arabidopsis (*Arabidopsis thaliana*) *tt7* seedlings overexpressing *F3*′*H* genes isolated from either apple or sorghum showed a restored ability to produce 3′,4′-hydroxylated flavonoids such as cyanidin and quercetin [82, 83]. The increases of these two flavonoids were also shown in transgenic tobacco (*Nicotiana tabacum*) overexpressing apple *F3′H* [83]. Overexpression of the ginkgo (*Ginkgo biloba*) gene *GbF3*′*H1* enhanced flavonoid production in transgenic poplar (*Populus davidiana*), as shown by the significantly increased red pigmentation and higher concentrations of epigallocatechin, gallocatechin, and catechin compared with that in the wild-type plants [90].

### Flavonoid 3′,5′-hydroxylase

F3′5′H (EC 1.14.13.88) is another key hydroxylase responsible for the B-ring hydroxylation of flavonoids and plays a crucial role in the formation of 3′,4′,5′-trihydroxylated derivatives (Fig. 2). Initially, *F3*′*5*′*H* was discovered in delphinidin-rich plants, responsible for the production of blue flower colors, and was therefore known as the blue gene [91]. Later, such genes were also identified widely in other plants that accumulate proanthocyanidins or flavonols possessing trihydroxyls on the B-ring. However, not all plant species have the ability to accumulate such B-ring trihydroxyflavonoids, indicating that the presence or expression of an appropriate *F3*′*5*′*H* gene is not ubiquitous in plants [92]. For example, there is no *F3*′*5*′*H* gene in the genome of the model plantarabidopsis.

To date, a number of *F3*′*5*′*H*s have been isolated from various horticultural crops, such as potato (*Solanum tuberosum*) [93], grapevine [81], tomato (*Solanum lycopersicum*) [94], pea (*Pisum sativum*) [95], and tea [96, 97]. Through heterologous expression in yeast, F3′5′Hs from tomato [94], tea [86, 96], grapevine [96] or montbretia [88] have been shown to hydroxylate the C3′ and/or C5′ positions of a broad range of flavonoid substrates, including flavanones, dihydroflavonols, flavonols, and/or flavones, with naringenin generally being the optimum substrate (Fig. 2). Thus, F3′5′H generally has a similar function to F3′H, and also possesses catalytic activity capable of converting naringenin or eriodictyol to 5,7,3′,4′,5′-pentahydroxy flavanone, dihydrokaempferol or dihydroquercetin to dihydromyricetin, and kaempferol or quercetin to myricetin, and also transforming luteolin into tricetin (Fig. 3).

In addition, expression levels of genes encoding F3′5′Hs greatly affect the accumulation of flavonoids with diverse B-ring hydroxylation patterns (Table 3). The red-skinned potato cultivar ‘Désirée’ transformed with a potato *F3*′*5*′*H* became purple-skinned, and the major anthocyanins were changed from pelargonidin derivatives to petunidin derivatives [93]. Silencing the *F3*′*5*′*H* in transgenic potato tuber impaired anthocyanin biosynthesis and caused a 100-fold increased level of kaempferol [98]. Similarly, *F3*′*5*′*H* mutants of pea have lost the ability to synthesize delphinidin and petunidin, which are the main pigments in wild-type pea flowers [95]. Additionally, transgenic petunia lines carrying *VvF3*′*5*′*H1* accumulate the 3′,4′,5′-hydroxylated-based anthocyanin malvidin, and show a shift from kaempferol to quercetin, compared with the non-transgenic control [81]. Overexpression of tea *F3*′*5*′*H* in tobacco plants caused the majority of transgenic flowers to become magenta, compared with the pale pink of the untransformed host, due to the accumulation of a novel 3′,4′,5′-hydroxylated-based anthocyanin, delphinin, and the cyanidin content was also increased [96]. So far, transgenic studies related to *F3*′*5*′*H* genes have focused preferentially on the biosynthesis of anthocyanins that confer the blue or purple colors of plant tissues.

### Flavanone 3-hydroxylase

F3H (EC 1.14.11.9) belongs to the FeII/2-ketoglutarate-dependent dioxygenase family. It catalyzes the 3β-hydroxylation of 2*S*-flavanones at the C3 position to 2*R,*3*R*-dihydroflavonols in the presence of O_2_, 2-oxoglutarate, Fe^2+^, and ascorbate as cofactors [99, 100] (Fig. 2). The first *F3H* was cloned from *Antirrhinum majus* [101], and subsequently additional *F3H*s were isolated from other plant-based foods, such as soybean (*G. max*), apple (*Malus domestica*), pear (*Pyrus communis*), *Lycium chinense*, and tea [102–105]. Heterologous expression of *Malus* or *Pyrus* F3H in yeast, and montbretia or tea F3H in *Escherichia coli*, showed these proteins could all catalyze the conversion of naringenin and eriodictyol into dihydrokaempferol and dihydroquercetin, respectively [88, 103, 105] (Fig. 3). Similarly, when LcF3H was expressed in *E. coli*, the recombinant enzyme showed the ability to convert naringenin to dihydrokaempferol [104]. Further, recombinant tea F3H was able to accept 5,7,3′,4′,5′-pentahydroxy flavanone as substrate to form dihydromyricetin [106]. Interestingly, in a heterologous assembly of the fisetin biosynthetic pathway in *E. coli*, the production of fisetin was achieved by the conversion of liquiritigenin into garbanzol as an intermediate under the catalytic action of F3H from arabidopsis [107] (Fig. 2).

F3H shares the flavanones as substrates with other enzymes involved in the synthesis of 3-deoxyflavonoids, and the dihydroflavonols it produces are the key biosynthetic precursors of flavonols, anthocyanins, and proanthocyanins [108]. Hence, altered expression levels of *F3H* might redirect the flavonoid accumulation patterns *in planta* (Table 3) and silencing of *F3H* in apple led to an accumulation of flavanones [109]. Transgenic tobacco overexpressing *F3H* from either tea or *L. chinense* showed significantly increased contents of flavanols such as catechin, epicatechin, and epigallocatechin [104, 110]. The *anthocyanin reduced* (*are*) tomato mutant has a mutation in the gene encoding F3H and accumulates higher concentrations of naringenin and lower levels of flavonols and anthocyanins, compared with those in the wild type. This phenotype was reversed after the complementation of *are* with the *p35S:F3H* transgene [111]. The seeds of transgenic arabidopsis carrying tea *F3H* showed markedly increased contents of kaempferol glycosides and oligomeric proanthocyanidins [105]. Additionally, as expected, transgenic expression of F3H in sorghum resulted in an enrichment in its flavonoid profile, and CRISPER/Cas9-mediated gene editing of F3H as well as flavone synthase (FNS) in soya bean significantly altered the accumulation of isoflavones, as shown by the doubling in leaf isoflavone content in the T_3_ generation of homozygous triple mutants compared with that of the wild type [112, 113].

### Flavone 6-hydroxylase

During the synthesis of the basic flavonoid skeleton catalyzed by CHS, the hydroxyl groups in the C5 and/or C7 positions are added to the A ring. It has also been found that additional hydroxylations can occur at other carbon sites on the A ring, such as C6 and C8, catalyzed by F6H and F8H, respectively (Fig. 2).

F6H was first identified from soybean and was shown to be a CYP450-dependent hydroxylase [114]. It efficiently catalyzes the hydroxylation at the C6 position of various flavanones but is hardly active with isoflavones. However, in soybean, there are several kinds of isoflavonoid constituents that possess a 6-hydroxyl group. Further investigation indicated that such 6-hydroxylation might occur before the 1,2-aryl migration of the flavonoid B ring in the process of isoflavanone formation [114]. Subsequently, a flavonol 6-hydroxylase was isolated and characterized from *Chrysosplenium americanum*, which exhibited 2-oxoglutarate-dependent dioxygenase (ODD) activity [115]. Meanwhile, Halbwirth *et al*. [116] identified another novel flavonol 6-hydroxylase, a microsomal CYP450-dependent monooxygenase in the petals of *Tagetes patula* and *Tagetes erecta* that could introduce a hydroxyl at the C6 of quercetin leading to the formation of quercetagetin. An F6H was also found in sweet basil (*Ocimum basilicum* L.) that could catalyze the 6-hydroxylation of flavones possessing 5-hydroxyl and 7-methoxyl residues [117]. Furthermore, another F6H was isolated from *S. baicalensis*, which was encoded by a CYP450 enzyme, namely SbCYP82D1.1 [118]. This can convert flavones without 7-*O*-methyl groups, such as apigenin and chrysin, into scutellarein and baicalein, respectively (Fig. 2). To prove the function of such SbF6H enzymes *in planta*, hairy root transformants in which *SbCYP82D1.1* was knocked down showed significantly reduced levels of baicalein and its glycosides baicalin but higher contents of chrysin glycosides. Also, arabidopsis overexpressing SbCYP82D1.1 accumulated baicalein [118] (Table 3). Moreover, an engineered *E. coli* that expressed diverse plant flavonoid biosynthetic pathway genes, including *SbF6H*, produced baicalein and scutellarein successfully, which further verified its 6-hydroxylation function [119] (Fig. 3). In addition, EbF6H, an F6H isolated from *Erigeron breviscapus*, also converted apigenin to scutellarein, which was demonstrated in a yeast cell factory [120] (Fig. 3).

### Flavone 8-hydroxylase

F8H was first isolated from the petals of *Chrysanthemum segetum* and shown to add a hydroxyl group at position 8 of flavonols and flavones, for instance, converting quercetin to 8-hydroxylquercetin (gossypetin) and luteolin to 8-hydroxyluteolin [103]. An F8H was subsequently identified from sweet basil trichomes and shown to be a specialized Rieske-type oxygenase that could efficiently catalyze the 8-hydroxylation of salvigenin [121]. Another CYP450 enzyme from *S. baicalensis*, SbCYP82D2, functions as an F8H with high substrate specificity that accepts only chrysin as substrate to produce norwogonin (Fig. 2), the precursor of wogonin, as shown by *in vivo* yeast assays [118] (Fig. 3). Further investigation in plants found that *SbCYP82D2*-silenced hairy roots of *S. baicalensis* showed decreased levels of wogonin and wogonoside, and transgenic arabidopsis overexpressing *SbCYP82D2* successfully accumulated norwogonin, which confirmed its 8-hydroxylation function [118] (Table 3). Interestingly, a novel F8H isolated from *S. barbata*, named SbarCYP82D-6, which has 62% amino acid identity with the above-mentioned F8H encoded by SbCYP82D2, was reported to show increased activity and produce substantial amounts of norwogonin from chrysin [122].

### Other hydroxylases

In addition to the common hydroxylases mentioned above, a few flavonoid substances also require the participation of other specialized hydroxylases, such as flavanone 2-hydroxylase (F2H). Flavone synthase II from *Medicago truncatula*, abbreviated as MtFNSII, converts flavanones to 2-hydroxyflavanone [123]. CYP93G2, a functional F2H, converts naringenin and eriodictyol to the corresponding 2-hydroxyflavanones required for C-glycosylflavone biosynthesis in rice [124]. However, according to the hydroxylation patterns of diverse flavonoid compounds (summarized in Fig. 2), there are some flavonoids whose hydroxylation mechanism has not yet been established, such as morin, which possesses an unusual C2′-OH, and galangin, a flavonol with no hydroxyl group on its B ring. Whether there are corresponding hydroxylases responsible for their formation needs to be investigated.

## Towards a biofactory scale-up for production of desirable flavonoids

The excellent bioactivities and biosafety of diverse natural flavonoids are attracting a great deal of attention for their potential applications in food products or as supplements with health-promoting properties. At present, commercially available flavonoids are mainly derived from plant sources. However, the low efficiency and high cost of the preparation of such flavonoids limits their exploitation. The biosynthesis of flavonoids in microorganisms with their mild reaction conditions and high yield is attracting considerable attention (Fig. 3). For instance, vectors constructed with either *F3H* or *FLS* from *Citrus* have both been introduced into *E. coli* and used to produce 15.1 mg l^−1^ kaempferol from tyrosine and 1.1 mg l^−1^ galangin from phenylalanine [125]. By overexpressing F3′H in *E. coli*, fisetin was synthesized at a concentration of 1.2 mg l^−1^ by supplementing with 0.05 mmol l^−1^ resokaempferol [107]. F3H and FLS introduced successfully into *Corynebacterium glutamicum* produced 23 mg l^−1^ kaempferol and 10 mg l^−1^ quercetin [126]. F6H and its partner P450 reductase were very effective for the production of both baicalein (8.5 mg l^−1^) and scutellarein (47.1 mg l^−1^) upon supplementation with 0.5 g l^−1^ phenylalanine and tyrosine in *E. coli* [119]. Furthermore, Appelhagen *et al*. [127] succeeded in developing and scaling up production of anthocyanins (90 mg l^−1^ cyanidin 3-*O*-rutinoside) in plant cell cultures that could be used as natural food colorants. It seems highly likely that additional applications will be developed as knowledge of flavonoid biosynthesis increases.

## Hydroxylation patterns and the degradation of natural flavonoids

Plant secondary metabolites such as flavonoids are not inert end products but they are subject to degradation in the plant [128]. The gradual decrease or complete disappearance of flavonoids observed in specific plant tissues demonstrates that catabolic turnover of flavonoids occurs generally. Myricetin, for example, was recently reported to accumulate to higher levels in younger fruits than in mature fruits of *Morella rubra* [129], although how it is degraded remains unknown. Thus, biosynthesis and further catabolism of flavonoids occur for both secondary constituents as well as primary metabolites.

There is, however, a quite limited amount of information reported describing the catabolism of flavonoids in plants. Decades ago, Barz and Koster [128] proposed that peroxidative chemistry might be involved in their catabolism. This hypothesis recently received support from investigations in arabidopsis and tomato, which showed that peroxidative cleavage of kaempferol contributed to the biosynthesis of the 4-hydroxybenzenoid moiety of the vital respiratory cofactor ubiquinone [130]. In contrast, there is a lot known about the degradation of flavonoids by microbes, which involves reactions such as dehydroxylation, deglycosylation, demethylation, decarboxylation, or isomerization carried out by rhizosphere microorganisms [131, 132] or gut microbiota [133].

Notably, the free hydroxyl group on C3 of kaempferol was proposed to be crucial for peroxidative cleavage of kaempferol [130]. Similarly, for degradation of plant flavonols by pirin proteins from *E. coli* or human, the 3-hydroxyl group of the ‘flavonol backbone’ was found to be important for the specific enzyme–substrate interaction [134]. For plant–pathogen interaction, *Sclerotinia sclerotiorum* was found to be able to catabolize flavonols through enzymes such as quercetin 2,3-dioxygenase [135]. Therefore, hydroxylation in key places on the ring may help render flavonoids ready to participate in degradation through the action of dioxygenases.

## Conclusions and perspective

Flavonoids with diverse hydroxylation patterns are important secondary metabolites and food constituents produced by plants during their development or as a defense against various environmental stresses. With the continued development of metabolomics and functional genomics, additional flavonoids are likely to be discovered in food plants with more diverse hydroxylation patterns generated by additional hydroxylases. It is anticipated that further structure–activity investigations, combined with a better understanding of regulatory mechanisms, will identify new bioactive compounds of great nutritional value. The rapid development of synthetic biology in the food and biomedicine industries, combined with new scale-up strategies, opens the prospect for mass production of desirable bioactive flavonoids. This is expected to play an important role in the amelioration and prevention of global high risk of non-communicable diseases. In addition, the recent discovery of ubiquinone biosynthesis from catabolic turnover of flavonoids *in planta* may open the door for new lines of inquiry.

## Acknowledgements

This work was supported by the Key Research and Development Program of Zhejiang Province (2021C02001), the National Natural Science Foundation of China (31872067), the Key Project for New Variety Breeding in Agriculture of Zhejiang Province (2021C02066-3), the 111 project (B17039), and the Fundamental Research Funds for the Central Universities.

## Author contributions

Y.L. and X.L. designed the review. Y.L., J.Q., J.L., and M.X. conducted the literature review and wrote the manuscript. D.G. and X.L. carefully compiled and revised the paper. C.S., C.X., and K.C. provided discussion and comments on the paper. All authors approved the final submission.

## Conflict of interest

The authors declare no conflict of interests.
